# Supplementary figures and images for: A comparative analysis of the dendritic cell response upon exposure to different rabies virus strains
Source: PLoS Negl Trop Dis. 2025 Apr 10;19(4):e0012994. doi: 10.1371/journal.pntd.0012994 (PMC12017532; doi:10.1371/journal.pntd.0012994)

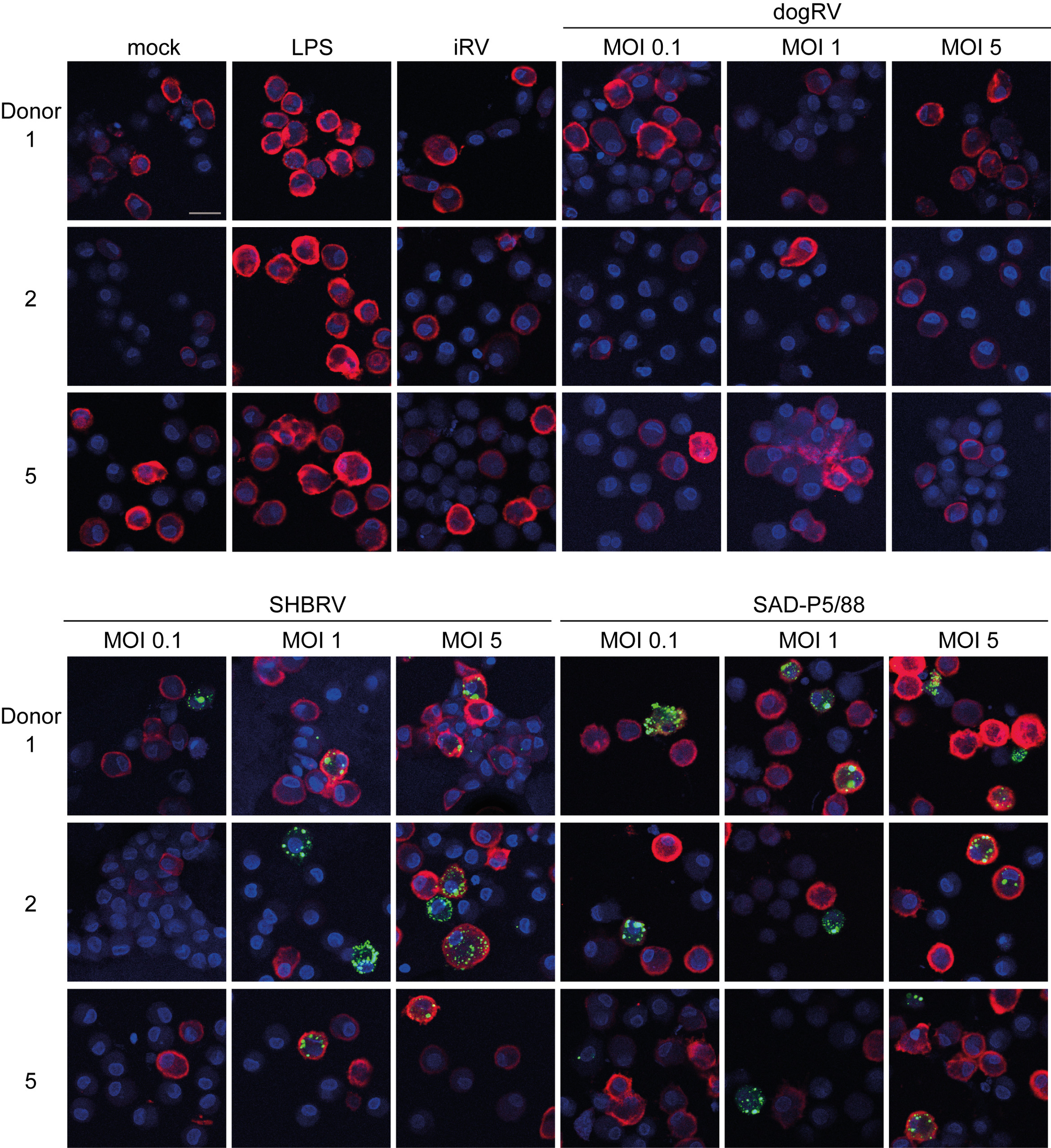

Supplement: S1 Fig — Immunofluorescence imaging of moDCs of n = 3 donors exposed to conditioned medium (mock), LPS, BPL-inactivated RABV (iRV), or either of the RABV strains dogRV, SHBRV, or SAD P5, at MOIs of 0.1, 1, and 5. moDCs were harvested 48 h p.i., fixed, and stained for intracellular RABV-N (green), CD86 (red), and nuclei (blue). Scalebar represents 20 µm. (TIF) [file pntd.0012994.s001.tif]

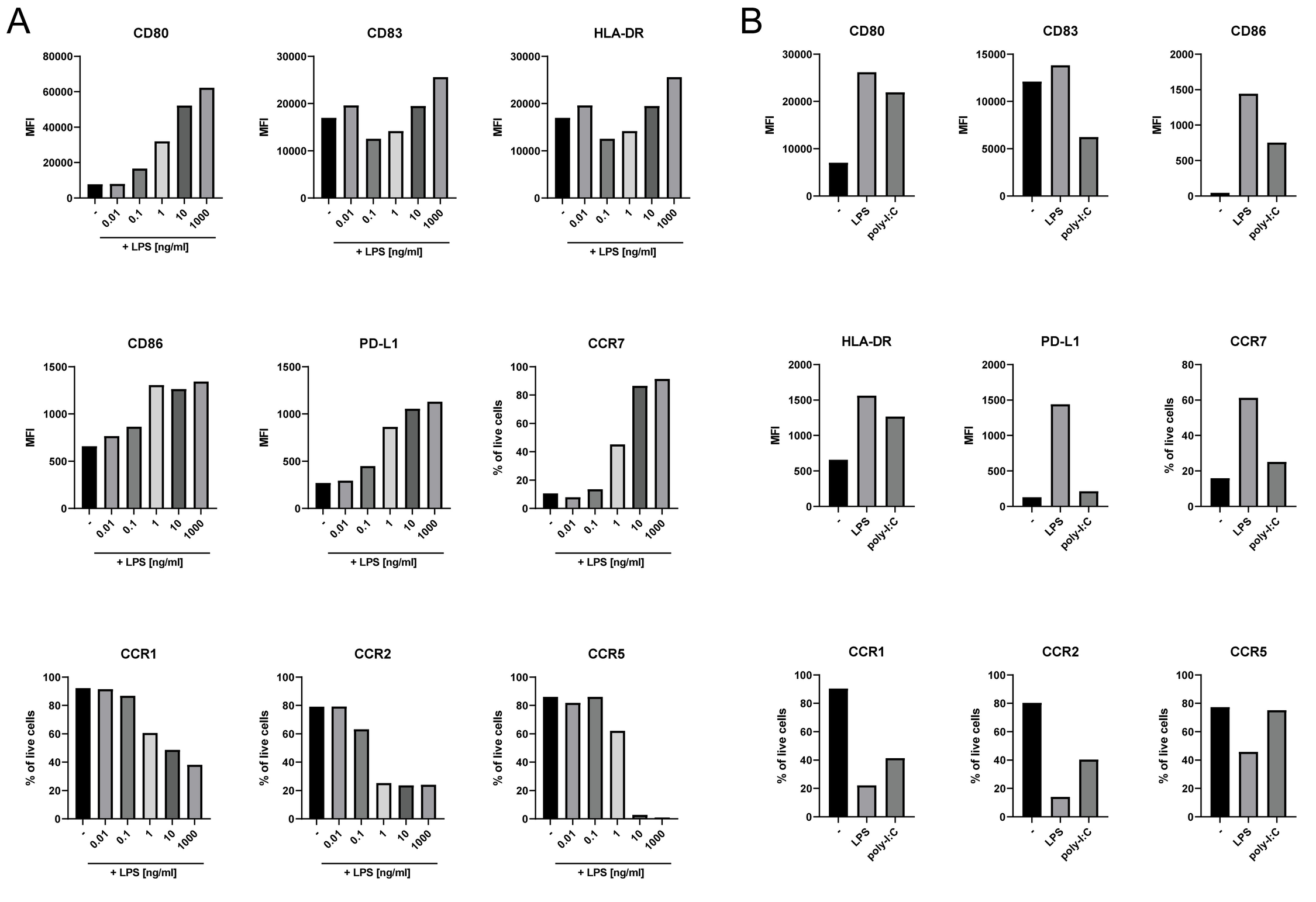

Supplement: S2 Fig — A Maturation and migration marker expression of moDCs in response to LPS in concentrations ranging from 0.01 ng/ml to 1000 ng/ml. B Maturation and migration marker expression of moDCs in response to stimulation with LPS (10 ng/ml) or poly-I:C (10 µg/ml). MFI – median fluorescence intensity. (TIF) [file pntd.0012994.s002.tif]

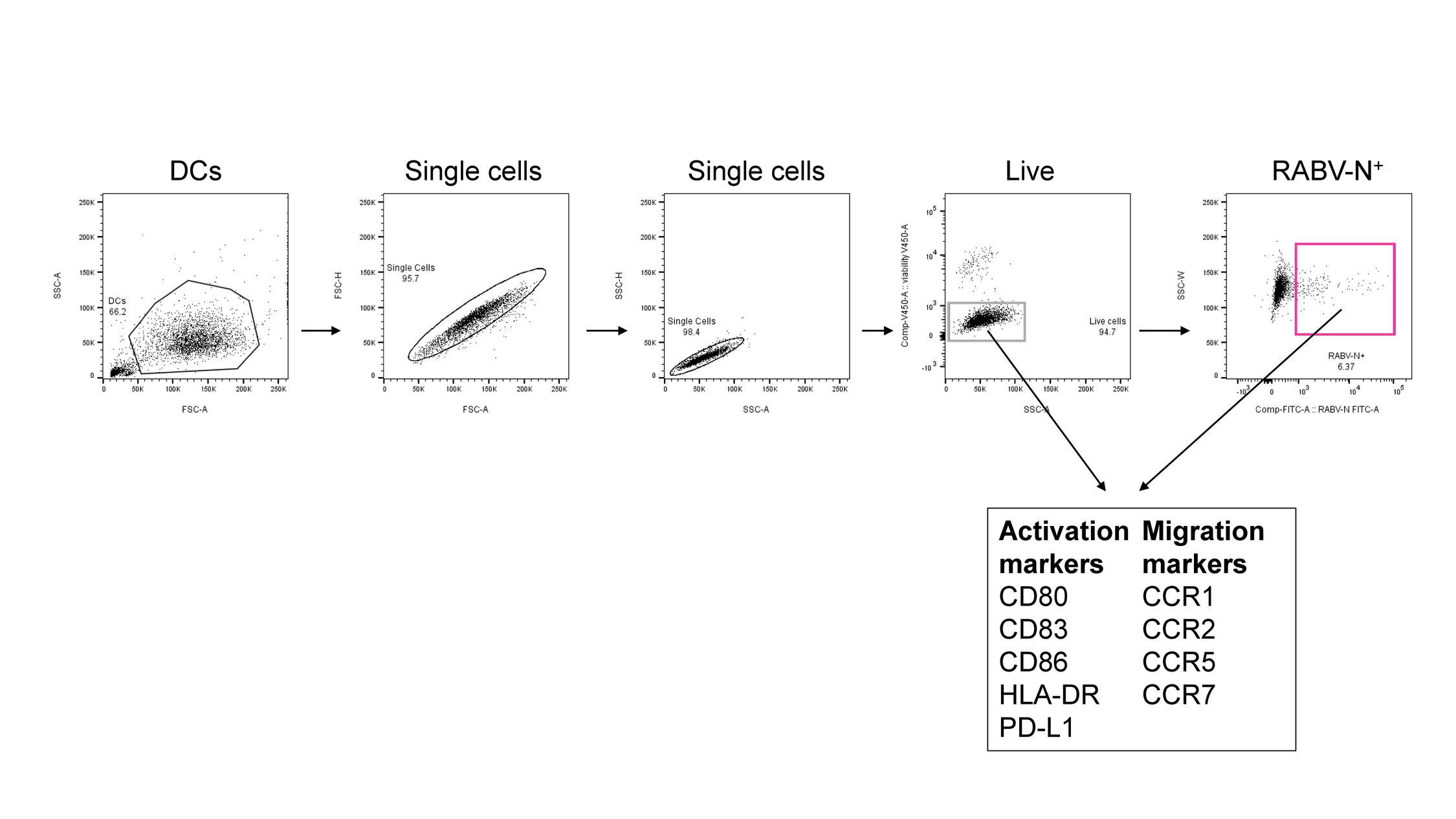

Supplement: S3 Fig — Surface activation and migration marker expression was measured by flow cytometry both within the overall live moDC fraction (grey) and the RABV-N+ fraction (pink). (TIF) [file pntd.0012994.s003.tif]

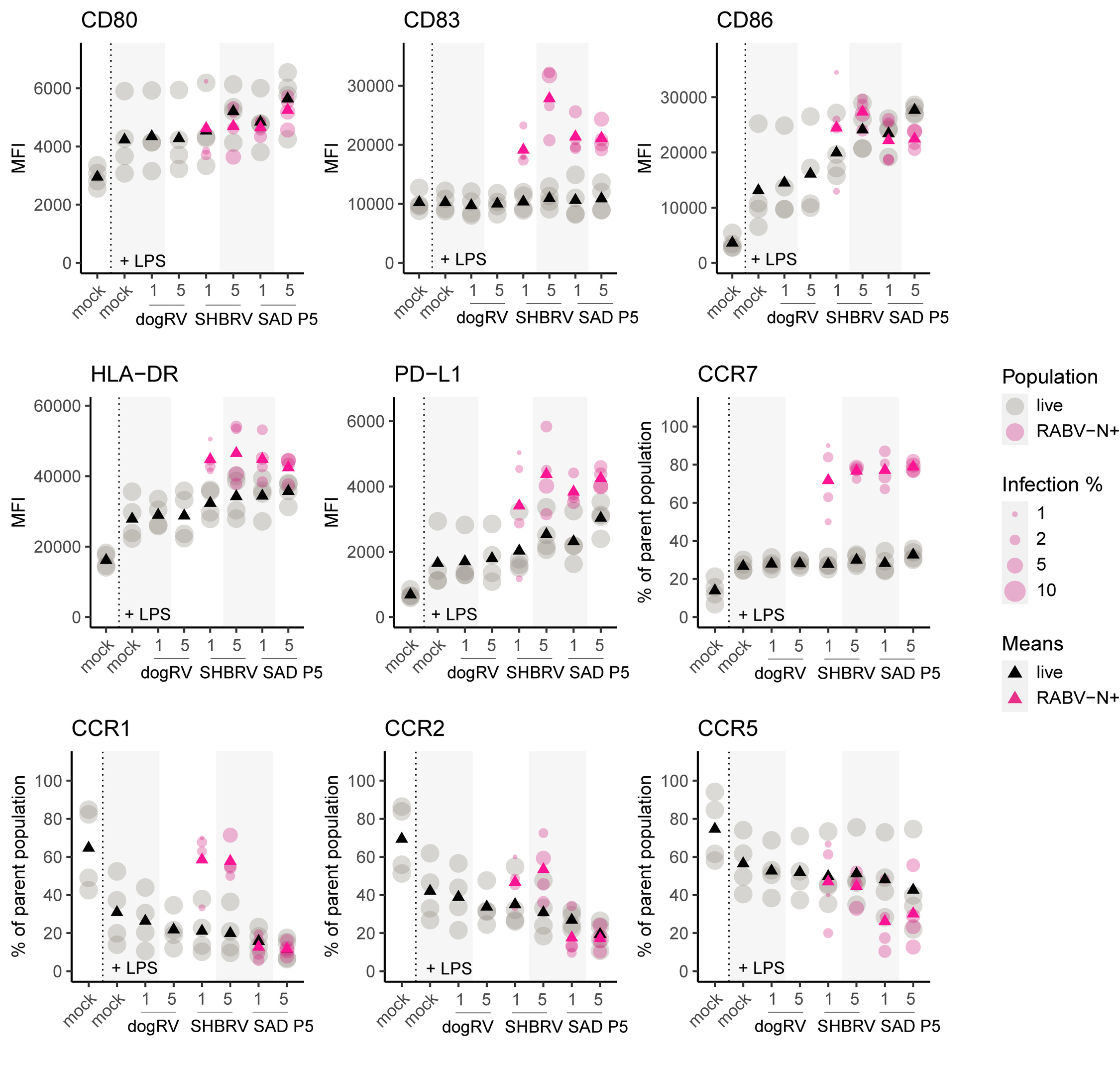

Supplement: S4 Fig — moDCs were exposed to RABV strains dogRV, SHBRV, and SAD P5, at MOIs of 1 and 5, treated with LPS at 24 h and analyzed 48 h post-treatment. Total live moDC fraction is depicted in grey and the infected (RABV-N+) fraction is depicted in pink. Triangles represent the mean values of the respective population. Dot sizes of the RABV-N+ fraction correspond to the percentage of infected cells. n = 4 individual donors. MFI – median fluorescence intensity. (TIF) [file pntd.0012994.s004.tif]
