# Supplementary material for: A comparative analysis of the dendritic cell response upon exposure to different rabies virus strains
Source: PLoS Negl Trop Dis. 2025 Apr 10;19(4):e0012994. doi: 10.1371/journal.pntd.0012994 (PMC12017532; doi:10.1371/journal.pntd.0012994)
Supplement: S1 Table — Measured by flow cytometry using intracellular staining of RABV-N protein. (DOCX) [file pntd.0012994.s005.docx]

**S1 Table. Infection percentages of moDCs exposed to SHBRV and SAD P5 RABV strains.** Measured by flow cytometry using intracellular staining of RABV-N protein.

| **RABV strain** | **MOI** | **% RABV-N+ moDCs** | | |
| --- | --- | --- | --- | --- |
|  |  | Donor 1 | Donor 2 | Donor 5 |
| SHBRV | 0.1 | 0.91 | 0.13 | 0.70 |
|  | 1 | 2.81 | 1.39 | 2.03 |
|  | 5 | 5.55 | 5.35 | 6.95 |
| SAD P5 | 0.1 | 0.071 | 0.24 | 0.17 |
|  | 1 | 1.4 | 2.43 | 1.10 |
|  | 5 | 6.75 | 10.9 | 4.77 |
