# Supplementary material for: A comparative analysis of the dendritic cell response upon exposure to different rabies virus strains
Source: PLoS Negl Trop Dis. 2025 Apr 10;19(4):e0012994. doi: 10.1371/journal.pntd.0012994 (PMC12017532; doi:10.1371/journal.pntd.0012994)
Supplement: Attachment — Submitted filename: 241128 Response to Reviewers.docx [file pntd.0012994.s008.docx]

**Point-by-point response to referees**

**Manuscript : *A comparative analysis of the dendritic cell response upon exposure to different rabies virus strains***

We thank the editor and reviewers for their time evaluating our manuscript and for the valuable and generous comments. We have incorporated changes to the manuscript and provided answers to all your comments and suggestions below. By doing so, we hope to have satisfactorily addressed all the issues and concerns. For clarity, the line numbers in the point-by-point response below refer to the lines in the clean (without track-changes) revised version of the manuscript.

***Reviewer #1***

*In the present study, the authors investigated the in vitro response of human monocyte-derived dendritic cells (moDCs) to different strains of rabies virus. The findings indicate that RABV strains exhibit variability in their capacity to infect and activate moDCs in vitro. Nevertheless, exposure to any RABV strain did not result in a notable antiviral status or the inhibition of moDC reactivity. This study offers insights into the manner in which rabies virus interacts with cells (DCs) that are pivotal for early immune responses, and underscores the necessity for further research to enhance immune activation by targeting dendritic cells. I believe that this study has implications for the field of rabies research; however, there are still some questions that require further investigation.*

*1. The three distinct RABV strains illustrated in Figure 1C were inoculated into moDC and SH-SY5Y cells. All three strains demonstrated successful infection with moDC at a multiplicity of infection (MOI) of 5, which yielded the highest viral titer. However, it is unclear why MOI 5 was not employed for infection with SH-SY5Y cells.*

We thank the reviewer for pointing out this inconsistency, and added a sentence in **line 121** for clarification. SH-SY5Y cells are highly susceptible to RABV infection and reach infection rates approaching 100 % at a low MOI of 0.1. Since they produce high titers of infectious virus even at lower MOIs, using an MOI > 1 for this cell line was considered unnecessary.

*2. The inability of moDCs exposed to RABV to elicit T cell proliferation in vitro is depicted in Figure 3. How was the underlying cause of the observed deficiency in T cell proliferation ascertained in this experiment? Was this caused by RABV infection, or caused by the RABV-treated DC cells?*

We agree that this is an important and interesting question. We did not investigate separate effects of RABV-infected and RABV-exposed moDCs on T cell proliferation. Firstly, because sorting of infected populations requires fixation for intracellular RABV antibody staining, which is incompatible with downstream functional assays. Secondly, because we expect a mixed moDC population to be present to interact with the naïve T cells in an *in vivo* infection setting. We added this argumentation in **lines 347-349.** Apart from that, we believe that any observed effect on T cell proliferation can be attributed mainly to the presence of moDCs (and not virus alone), since T cell activation requires co-stimulation (see e.g. Mattern et al., J Immunol 1994).

*3. This study primarily assessed the impact of various RABV strains on moDC. Should the authors evaluate the activation levels of MHC-I and MHC-II in response to different RABV strains following moDC treatment, in order to enhance the overall data quality?*

We agree with the reviewer that an extensive panel of additional markers, such as HLA-ABC (MHC-I), would be very interesting to investigate in targeted follow-up studies. To get an insight into moDC maturation, we chose a selection of markers that we considered relevant for antigen presentation (HLA-DR as a correlate of MHC-II) and co-stimulation (CD80, CD83, CD86), and that are commonly used to assess moDC activation. In addition, we chose to include markers for the moDC migration state in our flow cytometry panel.

***Reviewer #2:***

*The paper addresses a very important and meritorious question, as virus:host DC interactions are pivotal determinants of viral pathogenic strategies and host antiviral immunity. The studies are generally well executed and reach convincing, if limited, conclusions.*

*1. The extremely low infection rate in mDCs is a concern, hampering mechanistic analyses. The findings could indicate that DCs are not targeted by RV at all upon initial replication at the portal of entry, calling into the question the entire premise for the study. Viruses known to target DCs at the portal of entry (HIV, polio, dengue, VEE, etc.) readily will infect mDCs with high efficiency in vitro. At a minimum, the authors should discuss this possibility. In this reviewer’s opinion, the low infection rate will make it extremely difficult to reliably assess the RV:DC relationship in the mDC model.*

We agree that the infection percentages are very low, however, as RABV is a neurotropic virus, we were in fact surprised that moDCs were susceptible to RABV infection at all. The low infection percentages of moDCs could indeed indicate that moDCs are not targeted by RABV. This supports our hypothesis that a lack of moDC activation might be a viral immune evasion strategy during RABV infection.

In infections with, for example, HIV and dengue virus, the infection of DCs actively contributes to pathogenesis and viral transmission. This is a likely reason for the strong tropism of these viruses for moDCs, and affects moDC activation (St John & Rahore, Nat Rev Immunol 2019; van der Vlist et al., Curr Opin HIV AIDS 2011). On the other side, infection is not a pre-requisite to activate moDCs; in fact, infection of moDCs with SARS-CoV-2 and Influenza virus does not lead to productive infection but still leads to activation and an antiviral response (Wu et al., BMC Infectious Diseases 2011; Zheng et al., J Infect Dis. 2021). Furthermore, in previous experiments using human monocyte-derived macrophages, we did not observe any infection, but did observe a specific phenotype of macrophages in response to RABV exposure (Embregts et al., Front Immunol 2021).

Altogether, we do not think that the low infection rate of moDCs compromises our conclusions and we added this discussion in **lines 265-270.**

*2. The study lacks an investigation of the innate antiviral response. If RV targets DCs for infection, their ability to mount innate antiviral defenses should be investigated rigorously, eg. with detailed analyses of the innate signaling response.*

We agree that the study of the innate viral response is important, and it was explored by measuring cytokine release in supernatant (Fig 2 B). The panel that we used includes important cytokines of the innate antiviral response, such as type I, II, and type III IFNs. As this was not clearly described, we now emphasized this by adding text in the results section (**line 166**), as well as the discussion (**lines 316-321**). Although it is intriguing to investigate the activation of innate signaling pathways in response to RABV exposure, this was beyond the scope of this study.

*3. The authors use as simplistic concept of “DC activation” and its role in adaptive antiviral immunity. DC “activation” is not a switch that is turned on upon pathogen exposure, but rather a series of carefully orchestrated signaling responses that coordinate induction of maturation markers (eg.*

*CD80/86), LN homing receptors (eg. CCR7), the immuno-proteasome, peptide antigen trans-locators (TAP1/2), the peptide loading complex (tapasin:ERp57:MHC I), etc. These adaptive responses can lead to cross-presentation of viral antigen and priming of antiviral CD8 T cell immunity. Cross presentation/T cell priming are major targets for viral immune suppression strategies: even in circumstances where cytokines/maturation markers are induced (such as in the present case), other events (esp. antigen presentation/MHC I, DC viability) may be inhibited. The authors’ data on maturation markers/CCR7 induction in RV-infected mDCs indicate a certain activation state, but by no means suggests that these DCs are engaged in adaptive antiviral immunity (many other events important in cross presentation/T cell priming may be suppressed). This may explain that signs of DC engagement in vitro clashed with lacking DC function, eg. in LN homing/migration.*

We completely agree with the reviewer that DC activation requires *a series of carefully orchestrated signaling responses*. Here, we studied maturation and migration marker expression as well as cytokine release of the moDC response upon exposure to RABV, whereas antigen processing and cross-presentation are crucial as well in the induction of an effective T cell response. We therefore have now added lines on the discussion to refine the findings (**lines 341-349**).

*4. The comparisons with LPS are not helpful. LPS (TLR4 agonist), in the manner used here, is not a realistic or informative positive control for DC activation phenotypes. It is a “sledgehammer” inducer of innate signaling, with extreme, artificial, non-physiologic effects on myeloid cells. Since LPS exerts lethal toxicity in vivo, it is not a useful side-by-side comparator to more realistic, natural challenges of myeloid cells, such as (possible) RV infection.*

Indeed LPS induces extreme effects on the DCs, which we point out in **line 145.** We now included a new supplementary figure (**Fig S2**) to show the moDC response to different concentrations of LPS, supporting our choice to use a concentration of 10 ng/ml as positive control, since this concentration induced nearly the maximum response. In figure S2, we also included the moDC response to poly-I:C stimulation to show that a TLR ligand that more closely represents viral RNA is able to stimulate moDCs to a similar extent as LPS in our experimental setup.

For our main experiments, however, we specifically chose LPS instead of poly-I:C as positive control, since we expect LPS to be present at the bite site in natural RABV infections, as a component of the outer membrane of gram-negative bacteria. We added this explanation to **line 147.**

***Reviewer #3***

*This is a very interesting study by Kroh and colleagues, which explores the response to monocyte-derived DCs to Rabies virus infection using three different Rabies strains. The study has a lot of potential and very interesting results already, such as the fact that one of the strains does not infect DCs at all. However, there are some major issues in the opinion of this reviewer that require further insight. In its present form, I feel that the conclusions of the study are not supported by the data presented. Specific comments are as follows*:

*1- MoDCs represent only one subset of inflammatory DCs derived from monocytes (not from the pre-DC lineage) so, data cannot be extrapolated to DCs in general. The response of other DC subsets to Rabies virus infection may be completely different. For the same reasons, caution should be used by the authors when they extrapolate their findings to explain the T cell response to Rabies virus*

We agree with the reviewer and acknowledge that “DCs” is not the best definition to use throughout the manuscript. We therefore adjusted the manuscript and paid close attention to not use “DCs” when we only looked at moDCs. Furthermore we have now clarified why moDCS are of main interest for the current study **(lines 362-366).**

*2- The T-cell data is confusing for this reviewer. I understand from the methods section that the T cells utilized for the readout are autologous but not Rabies specific or even antigen-experienced T cell pools. I understand that these T cells proliferate in response to Dynabeads but I cannot expect these cells to proliferate in response to presentation of Rabies antigen if they are naïve, even if the moDCs are doing their job really well.*

*3- Related to this, the authors are exposing T cells to a mass population of moDCs in which, in the best case, only 10-15% of cells are infected. I know this is note easy, but what would happen if they used rabies infected sorted DCs?*

*4- For the reasons explained in point 2, I don´t think that the conclusion of Rabies virus activating moDCs but not leading to virus-specific T cell responses is correct. I think that, in order to conclude this, the authors would need to demonstrate that the Rabies exposed moDCs are not able to induce proliferation of Rabies-experienced T cells.*

We agree with the reviewers that it is hard to draw firm conclusions on the presented data. We therefore now have rephrased the respective paragraphs of the results and discussion sections, and put more focus on the T cell activation instead of T cell proliferation. We softened the conclusion that moDCs fail to activate the adaptive immune response as an extrapolation of our co-culture experiments. In addition, we made it more clear that we were looking at a naïve T cell population which reduces the extent of activation and proliferation that can be expected compared to using an antigen-specific T cell population. On the other hand, it most closely resembles the situation in the lymph nodes during natural infections. This is discussed in **lines 337-343.** Sorting of infected moDC populations is not possible, since the required fixing and permeabilization is incompatible with downstream functional analyses. In addition, this would not represent the natural situation with only a small percentage of infected moDCs. However, we would expect a stronger activation of T cells co-cultured with the infected compared to the uninfected moDC fraction, which we now discuss in **lines 347-349.**

***Minor***

*1- In the CFSE proliferation experiments it would be good to see the peaks of T cell proliferation (as a readout of CFSE dilution) rather than a bulk of cells that have lost CFSE fluorescence. That would help to compare and assess levels of T cell proliferation quantitatively.*

We agree that CFSE peaks show the more “classical” picture of proliferation, as compared to the T cell proliferation based on CFSE and side scatter (SSC) signals that we show in our manuscript. Unfortunately, in this experiment the CFSE dilution method did not result in clear proliferation peaks, and the population of proliferated T cells was very small. Thus, we chose to gate on proliferated and unproliferated T cells using the CFSE signal intensity, as well as the side scatter as a measure for T cell activation (see e.g. Bӧhmer et al., Cytometry 2011). We hope to clarify this approach by an addition in **line 192.**

*2- Is MFI median or mean fluorescence intensity? Median fluorescence intensity allows to better understand comparatively the levels of expression of the majority of cells in a mass population*

The information that MFI refers to median fluorescence intensity was added to figure captions of figure 2 and figure 4.

*3- LPS by itself stimulates T cell proliferation (see Tough et al., JEM 1997), so in Figure 3 any LPS carried out in the co-culture by the previous DC stimulation can do the job without DCs.*

The reviewer has raised an important point, and we cannot exclude that residual LPS was present in the co-cultures. However, we believe that any observed effect on T cell proliferation can be attributed mainly to the presence of moDCs, since T cell activation requires co-stimulation (see e.g. Mattern et al., J Immunol 1994).

*4- Can any virus expressing DC antagonist proteins suppress the stimulating effect of LPS?*

In Fig 4, we chose to show only the effect of LPS stimulation on the overall moDC population, and not the RABV-N+ (infected) cells. We now made this data available in **supplementary figure S4** and mention it in **line 220.** As in Fig 2, infected moDCs showed an even stronger response than the overall moDC population. We did not consider this relevant for the message that general RABV exposure does not suppress the ability of moDCs to respond to LPS stimulation, and for reasons of clarity only show the general population in the main figure 4.

References for viral proteins shown to suppress the response of DCs to LPS stimulation were added in **lines 353-355.**
